# Diagnosing Recurrent DVT of the Leg by Two Different Non–Contrast-Enhanced Magnetic Resonance Direct Thrombus Imaging Techniques: A Pilot Study

**DOI:** 10.1055/s-0039-1678683

**Published:** 2019-02-06

**Authors:** Charlotte E. A. Dronkers, Frederikus A. Klok, Kirsten van Langevelde, Alexandr Šrámek, Guido R. van Haren, Menno V. Huisman, Albert de Roos, Lucia J. M. Kroft

**Affiliations:** 1 Department of Thrombosis and Hemostasis, Leiden University Medical Center, Leiden, The Netherlands; 2 Department of Radiology, Leiden University Medical Center, Leiden, The Netherlands; 3 Department of Radiology, HAGA Teaching Hospital, The Hague, The Netherlands

**Keywords:** magnetic resonance imaging, venous thrombosis, diagnosis

## Abstract

**Introduction** Magnetic resonance direct thrombus imaging (MRDTI) is a promising technique to improve the diagnostic management of patients with a suspected ipsilateral recurrent deep vein thrombosis (DVT) by direct visualization of a thrombus. Another magnetic resonance imaging (MRI) technique, T1-weighted turbo spin-echo spectral attenuated inversion recovery (TSE-SPAIR), has the potential to image a thrombus directly with a high spatial resolution as well. The main aim of this pilot study was to investigate if adding the TSE-SPAIR sequence to an MRDTI sequence performed in patients with suspected recurrent DVT may increase the diagnostic confidence of expert MRDTI readers.

**Methods** Fifteen patients with suspected acute recurrent DVT were included in this study. The TSE-SPAIR sequence was scanned directly after the MRDTI scan but not used to guide clinical decision making, and both scans were adjudicated post hoc two times separately by three independent expert MRDTI readers. Diagnostic confidence was scored on a 4-point Likert scale: (1) poor (definite diagnosis impossible), (2) fair (evaluation of major findings possible), (3) good (definite diagnosis possible), and (4) excellent (exact diagnosis possible).

**Results** The diagnostic confidence of expert readers increased when adding the TSE-SPAIR sequence on top of the MRDTI sequence from “good” (median, 3.0; interquartile range [IQR], 2.66–3.0) to “excellent” (median, 3.67; IQR 3.33–3.67; *p* = 0.001). Evaluation of the scans in the reversed order 5 months after initial reading showed similar results. Diagnostic accuracy for proximal DVT of both scan techniques was good.

**Conclusion** The extra TSE-SPAIR sequence may help increase diagnostic confidence of radiologists in cases of uncertain diagnosis in patients with suspected ipsilateral recurrent DVT.

## Introduction

The diagnostic management of suspected ipsilateral recurrent proximal deep vein thrombosis (DVT) is complicated, mainly because of persistent intravascular abnormalities after a first DVT. 1 2 With the current imaging technique of first choice, i.e., compression ultrasonography (CUS), it is not always possible to make a distinction between residual vein thrombosis and acute recurrent DVT. CUS can only diagnose recurrent DVT with certainty in the case of a new noncompressible venous segment or an increase in vein diameter of a previously noncompressible vein when compared with a reference CUS assessed after a prior DVT. 3 4 In clinical practice, however, a reference CUS is often not available, making it impossible to diagnose an ipsilateral recurrent DVT with CUS. 5

Magnetic resonance imaging (MRI) is a noninvasive imaging technique. Several sequences have been evaluated for diagnosing DVT. Magnetic resonance direct thrombus imaging (MRDTI) is a T1-weighted gradient-echo sequence that is based on a shortened T1 signal after formation of methemoglobin in a fresh thrombus. 6 MRDTI has been shown to accurately diagnose a first DVT and can make a distinction between chronic residual thrombotic scars and an acute recurrent DVT. 7 8 9 MRDTI could therefore potentially be used in the diagnostic management of clinically suspected recurrent ipsilateral DVT. 2 10

In a pilot study, the T1-weighted turbo spin-echo spectral attenuated inversion recovery (TSE-SPAIR) sequence has been tested successfully in three patients with upper extremity DVT. 11 With this sequence, an acute thrombus can be directly visualized by the formation of methemoglobin in a fresh blood clot as well. Moreover, this sequence results in high-resolution images, with a particular clear resolution of the vessel wall. This latter quality has the potential to increase the accuracy of the diagnosis of ipsilateral recurrent DVT.

We hypothesized that this TSE-SPAIR sequence may have additional diagnostic value on top of the MRDTI sequence in diagnosing acute recurrent ipsilateral DVT.

## Methods

### Objectives

The objective of this pilot study was to assess the additional value of a TSE-SPAIR sequence on top of the standard MRDTI sequence with regard to diagnostic confidence, image quality, and diagnostic accuracy in the setting of suspected ipsilateral recurrent DVT. Diagnostic confidence was defined as the number of points on a 4-point Likert scale, specified as: (1) poor: definite diagnosis impossible, (2) fair: evaluation of major findings possible, (3) good: definite diagnosis possible, and (4) excellent: exact diagnosis possible. Image quality was scored on a 4-point Likert scale as well, specified as: (1) insufficient: insufficient for diagnosis, (2) adequate: adequate for diagnosis, (3) good: minimal inhomogeneity, and (4) excellent: no relevant artifacts. The results of the standard MRDTI sequence at baseline in combination with a 3-month follow-up period were used as the reference standard against which diagnostic accuracy (number of false-positive or false-negative tests) of the image analysis of the three independent reviewers was tested. Our secondary objective was to explore the diagnostic accuracy of the TSE-SPAIR sequence as single diagnostic test.

### Patients

Patients included in the Theia study at the Leiden University Medical Center (LUMC) between March and December 2016 were selected to participate in this study. The Theia study is a prospective, multicenter, single-arm management (cohort) study. 10 The primary objective of this study is to assess the safety of a negative MRDTI scan to rule out acute, recurrent ipsilateral DVT. Inclusion criteria for this study are: (1) suspected acute recurrent ipsilateral DVT as defined by a documented prior objectivated episode of DVT in the same leg as where the current symptoms originate from, (2) age ≥ 18 years, (3) ability of participant to understand the character and individual consequences of this study, and (4) signed and dated informed consent. Exclusion criteria are: (1) general contraindications for MRI, (2) CUS-proven acute symptomatic DVT within 6 months before current presentation, (3) onset of symptoms suggestive of acute recurrent DVT more than 10 days prior to presentation, (4) suspected acute pulmonary embolism, (5) hemodynamic instability at presentation, (6) medical or psychological condition that would not permit completion of the study or signing of informed consent, and (7) noncompliance or inability to adhere to treatment or follow-up visits. Patients on therapeutic anticoagulation were not excluded from the Theia study, since, in clinical practice, the diagnostic challenge in establishing the diagnosis of recurrent DVT is relevant to these patients as well. A confirmed diagnosis of recurrent DVT has important therapeutic consequences. The study protocol was approved by the local Institutional Review Board (P4.295, NL50663.058.14) and all patients provided written informed consent.

### MRDTI

In the context of the Theia study, patients underwent MRI within 24 hours after presentation with a suspected ipsilateral DVT with a 1.5T unit (Philips Ingenia 1.5T, release 5, Philips Medical Systems, Best, the Netherlands) with maximum gradient amplitude of 45 mT/m, slew rate of 200 T/m/s, using an integrated 16-channel posterior coil and a 16-channel anterior body coil for signal reception. For the MRDTI sequence, a T1-weighted magnetization-prepared three-dimensional gradient-echo sequence was used. The sequence includes a water-only excitation radiofrequency pulse (PROSET 121) to eliminate the fat signal, and the effective inversion time is chosen to nullify the blood signal. Scan parameters were: echo time (TE), 5.2 ms; repetition time (TR), 10 ms; turbo field echo prepulse inversion time, 1,200 ms; flip angle, 15 degrees; field of view, 400 × 362; acquisition resolution, 1.56 × 2.24 × 4 mm; 60 slices; slice thickness, 4 mm (acq)/2 mm (rec); slice gap, 0 mm. Imaging was performed on both legs simultaneously, from the ankle to the inferior vena cava in two or three imaging blocks, dependent on the length of the patient, using a 55-cm body coil. Scan time was 1.59 minutes for the MRDTI sequence per imaging block. In case of a positive MRDTI signal, anticoagulant treatment was started.

Patients with a negative MRDTI were subjected to a standardized CUS within 48 hours after initial presentation. This CUS served as reference test in case the patient returned with symptoms of ipsilateral recurrent DVT during follow-up; however, it was not used for management decisions at baseline. All patients were followed for 3 months for the occurrence of recurrent VTE.

### TSE-SPAIR Sequence

The TSE-SPAIR sequence was performed after the MRDTI sequence in the same session, without motion of the patient between the two scans. Three-dimensional TSE-SPAIR is a T1-weighted three-dimensional sequence using a spectral, adiabatic presaturation (inversion) pulse to achieve fat suppression. Scan parameters were: TE, 24 ms; TR, 400 ms; SPAIR inversion delay, 110 ms; flip angle, 90 degrees; field of view, 400 × 350; acquisition resolution 1 × 1 × 1 mm; 200 slices; slice thickness, 1 mm; slice gap, 0 mm. With this sequence, the affected leg was scanned from the calve to the hip in two imaging blocks, with an extra imaging time of 4.15 minutes per block, leading to a total imaging time of 20 minutes. This extra TSE-SPAIR sequence was not shown to the radiologist who evaluated the MRI scan for the Theia study. This way, the TSE-SPAIR sequence did not influence the final diagnosis and treatment of the patients.

### Image Analysis

After the follow-up period of the Theia study was completed for all patients included in the current analysis, the MRDTI and TSE-SPAIR sequences were evaluated separately by three independent readers with experience in MRDTI reading: K.v.L. (radiology resident with 5 years of experience), A.d.R. (radiologist with >20 years of experience), and A.S. (radiologist with 10 years of experience), who were blinded to the final clinical diagnosis and follow-up of the study subjects and had not seen the MRDTI images of the patients before the current reading. All scans were evaluated twice in two phases. First, the MRDTI scan was read and scored for diagnosis, diagnostic confidence, and image quality ( Table 1 ). Proximal DVT was defined as thrombus present in the external iliac, common femoral, deep femoral, superficial femoral, or popliteal vein, and distal DVT was defined as thrombus located below the knee in the posterior or anterior tibial, peroneal, or muscular veins. Second, the TSE-SPAIR sequence was shown to the readers, and the combination of the MRDTI and TSE-SPAIR scan was again scored for diagnosis, diagnostic confidence, and image quality. To be able to explore the diagnostic accuracy of the TSE-SPAIR sequence alone, all scans were scored for the second time by all reviewers, in the reversed order with a minimum period of 5 months in between the adjudications: first, the TSE-SPAIR sequence was scored for diagnosis, diagnostic confidence, and image quality, and thereafter, the MRDTI sequence was shown, and the combination of the TSE-SPAIR and MRDTI sequence was scored for the same variables.

**Table 1:** Scoring for diagnostic confidence and image quality: 4-point Likert scale 20

Diagnostic confidence MRDTI		Image quality MRDTI		Diagnostic confidence MRDTI + TSE-SPAIR/TSE-SPAIR + MRDTI		Image quality TSE-SPAIR	
(1) Poor	Definite diagnosis impossible	(1) Insufficient	Insufficient for diagnosis	(1) Poor	Definite diagnosis impossible	(1) Insufficient	Insufficient for diagnosis
(2) Fair	Evaluation of major findings possible	(2) Adequate	Adequate for diagnosis	(2) Fair	Evaluation of major findings possible	(2) Adequate	Adequate for diagnosis
(3) Good	Definite diagnosis possible	(3) Good	Minimal inhomogeneity	(3) Good	Definite diagnosis possible	(3) Good	Minimal inhomogeneity
(4)Excellent	Exact diagnosis possible	(4) Excellent	No relevant artifacts	(4) Excellent	Exact diagnosis possible	(4) Excellent	No relevant artifacts

### Statistics

It was predefined to include 15 patients in this study based on the number of patients included in comparable diagnostic studies. 12 13 The Wilcoxon signed-rank test was used to evaluate the difference in diagnostic confidence and image quality between the MRDTI and TSE-SPAIR sequence. To analyze overall differences, the Likert scale scores of the three independent readers were first combined and divided by 3. A *p* -value < 0.05 was considered statistically significant. The number of false-positive and false-negative tests based on the MRDTI sequence alone and MRDTI plus TSE-SPAIR sequences together, as well as the TSE-SPAIR alone and combined with MRDTI, were calculated based on the results of the majority, i.e., two out of three reviewers, with the clinical diagnosis based on MRDTI and events occurring during 3-month follow-up as reference. For example, when two reviewers evaluated MRDTI as positive for DVT and one as negative, and the results of the MRDTI scan at baseline including 3-month follow-up were negative for DVT, the test was reported as false positive. Exact diagnostic accuracy numbers, i.e., sensitivity and specificity, were not calculated since the sample size was too small. All statistics were performed using SPSS version 23 (IBM Corp, Armonk, NY).

## Results

### Patients

**Fig. 1:**
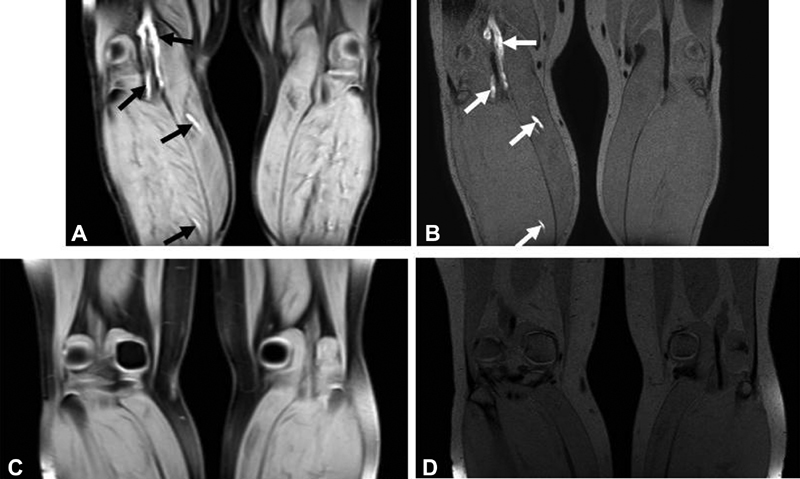
A 51-year-old male patient ( *patient 1* , Table 2 ) with a recurrent DVT of the left leg, shown on the MRDTI sequence ( **A** ) ( *black arrows* ) and TSE-SPAIR sequence ( **B** ) ( *white arrows* ). A 42-year-old female patient with a suspected recurrent DVT of the right leg ( *patient 11* , Table 2 ), ruled out based on the results of the MRDTI ( **C** ) and TSE-SPAIR ( **D** ) sequence.

**Fig. 2:**
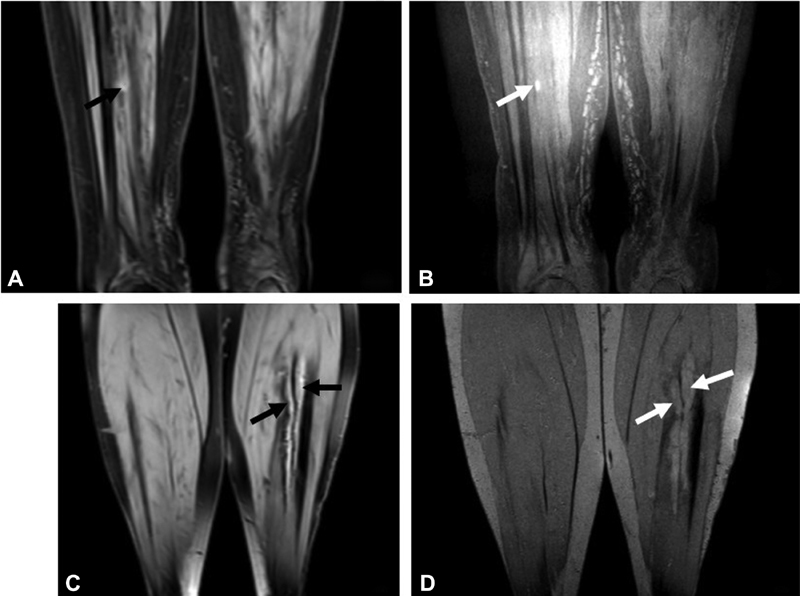
A 57-year-old man ( *patient 3* , Table 2 ) with a small peripheral calf vein thrombosis of the right leg, shown on the MRDTI sequence ( **A** ) ( *black arrow* ) and TSE-SPAIR sequence ( **B** ) ( *white arrow* ). A 70-year-old women ( *patient 7* , Table 2 ) with thrombosis in the calf veins, shown on the MRDTI sequence ( **C** ) ( *black arrows* ) and TSE-SPAIR sequence ( **D** ) ( *white arrows* ).

**Fig. 3:**
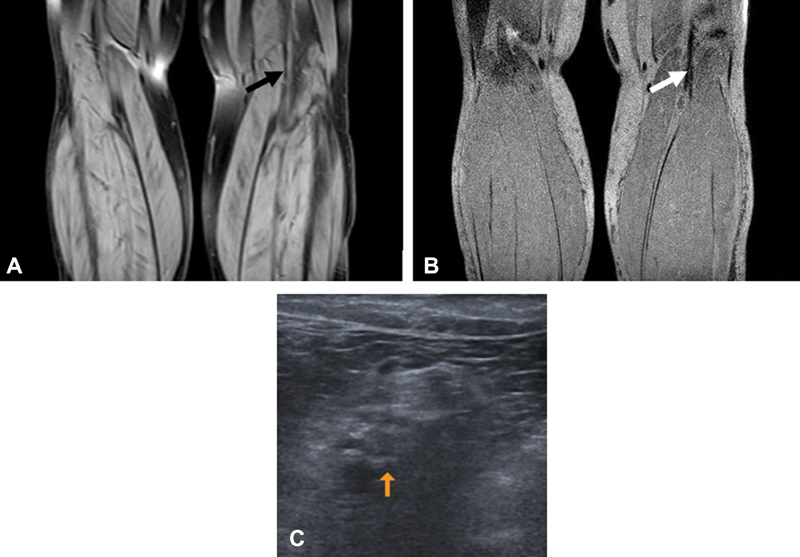
A 61-year-old man ( *patient 2* , Table 2 ) presenting with suspected recurrent DVT. MRDTI ( **A** ) and TSE-SPAIR sequence ( **B** ), showing no recurrent DVT in the left popliteal vein ( *black* and *white arrows* ). Compression ultrasound showed a small residual thrombus in the left distal popliteal vein ( **C** ) ( *orange arrow* ).

Of the 15 included patients, 8 were female and 7 male, and their mean age was 49 years (range, 24–71). The duration of complaints ranged from 1 to 10 days (median, 3; interquartile range [IQR], 2–8.5). Of the 15 patients, 6 patients used therapeutic anticoagulation at baseline. With MRDTI, one patient was diagnosed with proximal ipsilateral recurrent DVT ( Fig. 1 ) and anticoagulant therapy was started. Two patients were diagnosed with limited distal DVT, for which anticoagulant therapy was started as well ( Fig. 2 ). Twelve patients had a negative MRDTI scan, of which reference CUS was normal in seven. In the five remaining patients with a negative MRDTI scan, CUS indicated thrombus material in the femoral vein (one patient) or popliteal vein (four patients) ( Fig. 3 ). For none of these five patients, it was possible to make a distinction between acute DVT and residual DVT with CUS. None of the 12 patients with a negative MRDTI were treated with anticoagulants and none developed clinical symptoms of a recurrent VTE during a 3-month follow-up period.

### Diagnosis and Accuracy of MRDTI and MRDTI + TSE-SPAIR

Table 2 shows the diagnosis of the individual scans of each reviewer and the number of false-positive and false-negative tests of the three reviewers together based on the majority rule when comparing the MRDTI and MRDTI + TSE-SPAIR sequences against the results of the MRDTI sequence at baseline. The evaluation of the MRDTI sequences by the three independent readers corresponded overall for 93% with the original MRDTI reading. After adding the TSE-SPAIR sequence on top of the MRDTI sequence for evaluation, the consensus diagnosis changed for one patient, who two of three radiologists judged to have a normal MRDTI, to distal DVT based on the TSE-SPAIR. Notably, this patient was diagnosed with distal DVT and treated with anticoagulants based on the results of the MRDTI scan reading at baseline.

**Table 2:** Diagnosis and number of false-positive or false-negative cases for the majority of the three reviewers

Patient	Clinical diagnosis: based on MRDTI and events occurring during 3-month follow-up		MRDTI						MRDTI + TSE-SPAIR						TSE-SPAIR						TSE-SPAIR +MRDTI					
	Proximal DVT	Distal DVT	Proximal			Distal			Proximal			Distal			Proximal			Distal			Proximal			Distal		
			Reviewer			Reviewer			Reviewer			Reviewer			Reviewer			Reviewer			Reviewer			Reviewer		
			1	2	3	1	2	3	1	2	3	1	2	3	1	2	3	1	2	3	1	2	3	1	2	3
1	+	+	+	+	+	+	+	+	+	+	+	+	+	+	+	+	+	+	+	+	+	+	+	+	+	+
2	−	−	−	−	−	−	−	−	−	−	−	−	−	−	−	+	−	−	−	−	−	−	−	−	−	−
3	−	+	−	−	−	+	−	−	−	−	−	+	−	+	−	−	−	+	+	+	−	+	−	+	+	+
4	−	−	−	−	−	−	−	−	−	−	−	−	−	−	−	−	−	−	+	−	−	−	−	−	−	−
5	−	−	−	−	−	−	−	−	−	−	+	−	−	−	−	−	−	−	−	−	−	−	−	−	−	−
6	−	−	−	−	−	−	−	−	−	−	−	−	−	−	−	−	−	−	−	−	−	−	−	−	−	−
7	−	+	−	−	−	+	+	+	−	−	−	+	+	+	−	−	−	+	−	+	−	−	−	+	+	+
8	−	−	−	−	−	−	−	−	−	−	−	−	−	+	−	−	−	−	+	+	−	−	−	−	+	−
9	−	−	−	−	−	−	−	−	−	−	−	−	+	−	−	−	−	−	+	−	−	−	−	−	−	−
10	−	−	−	−	−	−	−	−	−	−	−	−	−	−	−	−	−	−	−	−	−	−	−	−	−	−
11	−	−	−	−	−	−	−	−	−	−	−	−	−	−	−	−	−	−	−	−	−	−	−	−	−	−
12	−	−	−	−	−	−	−	−	−	−	−	−	−	−	−	−	−	−	−	−	−	−	−	−	−	−
13	−	−	−	−	+	−	−	−	−	−	−	−	−	−	−	−	−	−	−	−	−	−	−	−	−	−
14	−	−	−	−	−	−	−	−	−	−	−	−	−	−	−	−	−	−	−	−	−	−	−	−	−	−
15	−	−	−	−	−	−	−	−	−	−	−	−	−	−	−	−	−	+	−	+	−	−	−	−	−	+
Number of false-positive and false-negative interpretations counted for the majority of the three reviewers						1 false-negative												2 false-positive								

Note: −, negative for DVT; +, positive for DVT.

### MRDTI and TSE-SPAIR Analysis: Diagnostic Confidence

The diagnostic confidence of MRDTI was scored overall, after pooling the results from the reviewers, as “good” for a Likert scale score 3.0 (IQR, 2.66–3.0). After adding the TSE-SPAIR sequence, the overall median diagnostic confidence increased significantly to “excellent” for a Likert scale of 3.67 (IQR, 3.33–3.67; *p* = 0.001) ( Table 3 ).

**Table 3:** Overall diagnostic confidence MRDTI and MRDTI + TSE-SPAIR sequences and vice versa

	Overall
Diagnostic confidence MRDTI	Median: 3.0 (IQR, 2.66–3.0)
Diagnostic confidence MRDTI + TSE-SPAIR	Median: 3.67 (IQR, 3.33–3.67)
Wilcoxon signed-rank test	*p* = 0.001
Diagnostic confidence TSE-SPAIR	Median: 3.33 (IQR, 3.0–3.67)
Diagnostic confidence TSE-SPAIR + MRDTI	Median: 3.67 (IQR, 3.0–3.67)
Wilcoxon signed-rank test	*p* = 0.176

Abbreviation: IQR, interquartile range.

### MRDTI and TSE-SPAIR Analysis: Image Quality

Overall, the image quality of the TSE-SPAIR sequence was judged to be slightly better than the MRDTI sequence, with a median increase from 2.67 (IQR, 2.67–3.00) to 3.00 (IQR, 3.00–3.33; *p* = 0.018) ( Table 4 ). There was no correlation between image quality and diagnostic confidence.

**Table 4:** Overall image quality of MRDTI and TSE-SPAIR

	Overall
Image quality MRDTI	Median: 2.67 (IQR, 2.67–3.00)
Image quality TSE-SPAIR	Median: 3.00 (IQR, 3.00–3.33)
Wilcoxon signed-rank test	*p* = 0.018
Image quality TSE-SPAIR	Median: 3.33 (IQR, 3.0–3.33)
Image quality MRDTI	Median: 3.0 (IQR, 3.0–3.33)
Wilcoxon signed-rank test	*p* = 0.35

Abbreviation: IQR, interquartile range.

### Evaluation in Reversed Order: TSE-SPAIR Alone and TSE-SPAIR + MRDTI

The evaluation of the TSE-SPAIR sequences alone corresponded overall for 82% with the original MRDTI reading ( Table 2 ). Based on the TSE-SPAIR sequence alone, two additional patients were diagnosed with distal DVT, on top of the three patients diagnosed with proximal or distal DVT on the aggregate reading of MRDTI. Indeed, after adding the MRDTI sequence, these two patients were adjudicated as not having DVT, which corresponded with the results of the MRDTI scan reading and management decision at baseline. Overall, the median diagnostic confidence increased after evaluation of the MRDTI sequence on top of the TSE-SPAIR sequence from a median of 3.33 (IQR, 3.0–3.67) to 3.67 (IQR, 3.0–3.67), although this change was not significant ( *p* = 0.176). Image quality of the TSE-SPAIR sequence was scored slightly better ( Table 4 ).

## Discussion

This study shows that after adding the TSE-SPAIR sequence on top of the MRDTI sequence for diagnosing proximal ipsilateral recurrent DVT, diagnostic confidence increased overall from “good” (median, 3.0; IQR, 2.66–3.0) to “excellent” (median, 3.67; IQR, 3.33–3.67). When the scans were evaluated in the reversed order, the diagnostic confidence did also increase but to a lesser extent. The image quality of the TSE-SPAIR sequence was scored higher than the MRDTI sequence for the first and second evaluation. The diagnostic accuracy of both imaging tests for proximal DVT was good, although the small sample size does not allow calculation and comparison of sensitivity.

With the MRDTI sequence, a thrombus can be visualized directly as a hyperintense signal based on the shorter T1 relaxation time in comparison with blood. The change in the T1 relaxation time is caused by change in paramagnetic properties by accumulation of methemoglobin in fresh thrombus, which is formed from hemoglobin by the oxidation of Fe ^2+^ into Fe ^3+^ during the acute phase of DVT. 6 14 Previous studies have shown that this high signal appears within 3 hours after thrombus formation and resolves completely after 6 months. 6 The same bright signal intensity can be visualized with the TSE-SPAIR sequence. Additionally, this sequence has a better spatial resolution, leading to improved visualization of the vessel wall. 13 The better visualization of the vessel wall may help (double) confirm that increased signal intensity is indeed present in one of the deep veins, and is not an artifact. In the MRDTI sequence, the signal in arteries may appear high due to inflow effect, even when using a saturation slab. This may explain the overall increase in diagnostic confidence for the reviewers when the TSE-SPAIR sequence was evaluated on top of the MRDTI sequence in this study. This raises the question whether TSE-SPAIR may be standardly combined to MRDTI scanning, and/or has the potential to be used as a single test, instead of MRDTI. Importantly, for the TSE-SPAIR sequence, the timing of development and disappearance of high signal intensity in thrombus is not validated yet. This was also noticed during evaluation of the TSE-SPAIR sequences alone: some venous segments were detected with an intermediate/“gray” signal and considered positive for DVT in two patients, although they were considered negative for DVT on the initial MRDTI evaluation. Of note and in contrast to MRDTI, TSE-SPAIR has to be tested against the gold standard in a larger patient group before it can be used as single test. An advantage of using the MRDTI and TSE-SPAIR sequences is that these are both non–contrast-enhanced MRI sequences. Disadvantages of using gadolinium-based contrast agents are costs of the contrast agent itself, application materials, and, more importantly, the extra time needed for contrast administration. Potential risks are adverse events (contrast allergy and nephrogenic systemic sclerosis in patients with severe renal insufficiency). 15 Also, recent research has suggested the potential risk of gadolinium retention in the human body. 16

A disadvantage of using the TSE-SPAIR sequence on top of the MRDTI sequence is the significant increase in MRI scan time, about 8 minutes per investigation. With the high costs and low availability of MRI time, this may be an important issue.

Similar non–contrast-enhanced direct thrombus MRI techniques have been investigated for the visualization of the intracranial arteries, the carotid artery, and the superficial femoral arteries. 17 18 19 To our knowledge, there is only one other study that investigated an MRI technique that is comparable with TSE-SPAIR for diagnosing DVT. 13 That was a small pilot study including 13 patients with a CUS-proven first DVT and using a 3.0T scanner with a volumetric isotropic turbo spin-echo acquisition technique (VISTA). Accuracy was calculated compared with contrast-enhanced MRI and ultrasonography as a reference standard. This resulted in a sensitivity of 77.8%, specificity of 94.8%, a negative predictive value of 91.6%, and a positive predictive value of 85.4%. Image quality and diagnostic confidence level of VISTA were 3.54 and 3.80, respectively, both scored on a 4-point Likert scale. Compared with our study, that study included patients with a suspected first DVT, instead of recurrent DVT in our study, and they used a contrast-enhanced MRI and ultrasonography as reference to the VISTA technique instead of another T1-weighted MRI technique. Additionally, the diagnostic confidence was based on the VISTA technique alone instead of MRDTI +TSE-SPAIR together in our study; therefore, direct comparison of the results of the two studies is not possible. However, the diagnostic confidences of VISTA and MRDTI + TSE-SPAIR were comparable with Likert scale scores of 3.80 and 3.67, respectively.

A limitation of our study was that we have no reference test to identify false-positive patients since it is not possible to differentiate residual thrombosis from acute recurrent thrombosis neither with ultrasound nor with venography. This indicated that the specificity of MRDTI was considered to be 100%, and the sensitivity of MRDTI could only improve if a patient with a negative MRDTI was diagnosed with a recurrence in the follow-up period. A second limitation was that the three independent readers were experienced in reading the MRDTI sequences, but had no experience in reading TSE-SPAIR sequences, which may have influenced our findings. Third, our sample size was small, most of them were negative cases, and the study was neither powered nor designed to compare the diagnostic accuracy of both scan sequences accurately. Lastly, some patients were treated with anticoagulants, which could have masked a false-negative diagnosis.

In conclusion, this pilot study shows that the diagnostic confidence improved when adding the TSE-SPAIR sequence to the MRDTI sequence for diagnosing acute recurrent proximal DVT. The extra (TSE-SPAIR) sequence may help increase diagnostic confidence in case of uncertain diagnosis.

## References

[1] Piovella F Crippa L Barone M Normalization rates of compression ultrasonography in patients with a first episode of deep vein thrombosis of the lower limbs: association with recurrence and new thrombosis Haematologica 2002 87 05 515 522 12010666

[2] van der Hulle T Dronkers C E Huisman M V Klok F A Current standings in diagnostic management of acute venous thromboembolism: Still rough around the edges Blood Rev 2016 30 01 21 26 26233662 10.1016/j.blre.2015.07.002

[3] Prandoni P Lensing A W Bernardi E Villalta S Bagatella P Girolami A; DERECUS Investigators Group.The diagnostic value of compression ultrasonography in patients with suspected recurrent deep vein thrombosis Thromb Haemost 2002 88 03 402 406 12353067

[4] Dronkers C E Klok F A Huisman M V Current and future perspectives in imaging of venous thromboembolism J Thromb Haemost 2016 14 09 1696 1710 27397899 10.1111/jth.13403

[5] Tan M Velthuis S I Westerbeek R ECJ Van Rooden C J Van der Meer F J Huisman M V High percentage of non-diagnostic compression ultrasonography results and the diagnosis of ipsilateral recurrent proximal deep vein thrombosis J Thromb Haemost 2010 8 04 848 850 20398187 10.1111/j.1538-7836.2010.03758.x

[6] Saha P Andia M E Modarai B Magnetic resonance T1 relaxation time of venous thrombus is determined by iron processing and predicts susceptibility to lysis Circulation 2013 128 07 729 736 23820077 10.1161/CIRCULATIONAHA.113.001371 PMC3983557

[7] Fraser D G Moody A R Morgan P S Martel A L Davidson I Diagnosis of lower-limb deep venous thrombosis: a prospective blinded study of magnetic resonance direct thrombus imaging Ann Intern Med 2002 136 02 89 98 11790060 10.7326/0003-4819-136-2-200201150-00006

[8] Tan M Mol G C van Rooden C J Magnetic resonance direct thrombus imaging differentiates acute recurrent ipsilateral deep vein thrombosis from residual thrombosis Blood 2014 124 04 623 627 24928859 10.1182/blood-2014-04-566380

[9] van Langevelde K Srámek A Vincken P W van Rooden J K Rosendaal F R Cannegieter S C Finding the origin of pulmonary emboli with a total-body magnetic resonance direct thrombus imaging technique Haematologica 2013 98 02 309 315 22801962 10.3324/haematol.2012.069195 PMC3561441

[10] Klok F A Tan M Huisman M V Letter by Klok et al regarding article, “18F-fluorodeoxyglucose positron emission tomography/computed tomography enables the detection of recurrent same-site deep vein thrombosis by illuminating recently formed, neutrophil-rich thrombus” Circulation 2015 131 24 e530 26078374 10.1161/CIRCULATIONAHA.114.013786

[11] Dronkers C EA Klok F A van Haren G R Diagnosing upper extremity deep vein thrombosis with non-contrast-enhanced magnetic resonance direct thrombus imaging: a pilot study Thromb Res 2018 163 47 50 29353683 10.1016/j.thromres.2018.01.015

[12] Schmitz S A O'Regan D P Gibson D Technical report: Magnetic resonance direct thrombus imaging at 3 T field strength in patients with lower limb deep vein thrombosis: a feasibility study Clin Radiol 2006 61 03 282 286 16488211 10.1016/j.crad.2005.10.009

[13] Treitl K M Treitl M Kooijman-Kurfuerst H Three-dimensional black-blood T1-weighted turbo spin-echo techniques for the diagnosis of deep vein thrombosis in comparison with contrast-enhanced magnetic resonance imaging: a pilot study Invest Radiol 2015 50 06 401 408 25783228 10.1097/RLI.0000000000000142

[14] Blume U Orbell J Waltham M Smith A Razavi R Schaeffter T 3D T(1)-mapping for the characterization of deep vein thrombosis MAGMA 2009 22 06 375 383 19946791 10.1007/s10334-009-0189-8

[15] Brockow K Sánchez-Borges M Hypersensitivity to contrast media and dyes Immunol Allergy Clin North Am 2014 34 03 547 564 25017677 10.1016/j.iac.2014.04.002

[16] Dekkers I A Roos R van der Molen A J Gadolinium retention after administration of contrast agents based on linear chelators and the recommendations of the European Medicines Agency Eur Radiol 2018 28 04 1579 1584 29063255 10.1007/s00330-017-5065-8

[17] Fan Z Zhang Z Chung Y C Carotid arterial wall MRI at 3T using 3D variable-flip-angle turbo spin-echo (TSE) with flow-sensitive dephasing (FSD) J Magn Reson Imaging 2010 31 03 645 654 20187208 10.1002/jmri.22058 PMC2841222

[18] Qiao Y Steinman D A Qin Q Intracranial arterial wall imaging using three-dimensional high isotropic resolution black blood MRI at 3.0 Tesla J Magn Reson Imaging 2011 34 01 22 30 21698704 10.1002/jmri.22592

[19] Zhang Z Fan Z Carroll T J Three-dimensional T2-weighted MRI of the human femoral arterial vessel wall at 3.0 Tesla Invest Radiol 2009 44 09 619 626 19692844 10.1097/RLI.0b013e3181b4c218 PMC2843396

[20] Likert R A technique for the measurement of attitudes Arch Psychol 1932 140 1 55

